# Endothelin@25 – new agonists, antagonists, inhibitors and emerging research frontiers: IUPHAR Review 12

**DOI:** 10.1111/bph.12874

**Published:** 2014-11-24

**Authors:** J J Maguire, A P Davenport

**Affiliations:** 1 Clinical Pharmacology Unit, University of Cambridge, Addenbrooke's HospitalCambridge, UK

## Abstract

Since the discovery of endothelin (ET)-1 in 1988, the main components of the signalling pathway have become established, comprising three structurally similar endogenous 21-amino acid peptides, ET-1, ET-2 and ET-3, that activate two GPCRs, ET_A_ and ET_B_. Our aim in this review is to highlight the recent progress in ET research. The ET-like domain peptide, corresponding to prepro-ET-1_93–166_, has been proposed to be co-synthesized and released with ET-1, to modulate the actions of the peptide. ET-1 remains the most potent vasoconstrictor in the human cardiovascular system with a particularly long-lasting action. To date, the major therapeutic strategy to block the unwanted actions of ET in disease, principally in pulmonary arterial hypertension, has been to use antagonists that are selective for the ET_A_ receptor (ambrisentan) or that block both receptor subtypes (bosentan). Macitentan represents the next generation of antagonists, being more potent than bosentan, with longer receptor occupancy and it is converted to an active metabolite; properties contributing to greater pharmacodynamic and pharmacokinetic efficacy. A second strategy is now being more widely tested in clinical trials and uses combined inhibitors of ET-converting enzyme and neutral endopeptidase such as SLV306 (daglutril). A third strategy based on activating the ET_B_ receptor, has led to the renaissance of the modified peptide agonist IRL1620 as a clinical candidate in delivering anti-tumour drugs and as a pharmacological tool to investigate experimental pathophysiological conditions. Finally, we discuss biased signalling, epigenetic regulation and targeting with monoclonal antibodies as prospective new areas for ET research.

**Table:** Tables of Links

TARGETS
**GPCRs***^a^*
AT_1_ receptors
ET_A_ receptors
ET_B_ receptors
GPR37
GPR37L
Hydroxycarboxylic acid receptors
μ opioid receptors
**Enzymes***^b^*
Cathepsin A
Chymase
CYP3A4
CYP2C19
Endothelin-converting enzyme 1
Endothelin-converting enzyme 2
Neutral endopeptidase

These Tables list protein targets and ligands that are hyperlinked to corresponding entries in http://www.guidetopharmacology.org, the common portal for data from the IUPHAR/BPS Guide to PHARMACOLOGY (Pawson *et al*., 2014) and the Concise Guide to PHARMACOLOGY 2013/14 (*^a,b^*Alexander *et al*., 2013a,b[Bibr b3],[Bibr b4]).

**Table:** 

LIGANDS	
5-Fluorouracil	Endothelin-3
Ambrisentan	IRL1620
Amyloid β-peptide	KC-12615
Atrial natriuretic peptide (ANP)	Losartan
β-Catenin	Macitentan
Bosentan	NO
BQ123	Prosaptide
BQ788	PGI_2_
BQ3020	Sarafotoxin S6b
Captopril	Sarafotoxin S6c
Daglutril	Sitaxentan
Docetaxel	TGFβ1
Doxorubicin	
Endothelin-1	
Endothelin-2

This article, written by members of the International Union of Basic and Clinical Pharmacology Committee on Receptor Nomenclature and Drug Classification (NC-IUPHAR) subcommittee for the endothelin receptors, confirms the existing nomenclature for these receptors and reviews our current understanding of their structure, pharmacology and functions, and their likely physiological roles in health and disease. More information on these receptor families can be found in the Concise Guide to PHARMACOLOGY (http://onlinelibrary.wiley.com/doi/10.1111/bph.12445/abstract) and for each member of the family in the corresponding database http://www.guidetopharmacology.org/GRAC/FamilyDisplayForward?familyId=21&familyType=GPCR.

## Introduction

**Figure 1:**
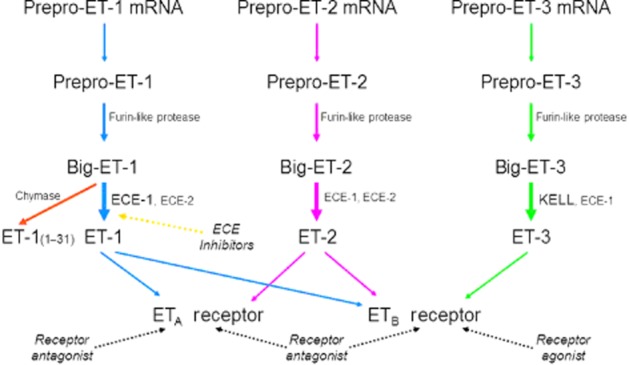
Scheme of the biosynthesis of ET peptides and their interaction with receptors. Based on information from the literature including Barton and Yanagisawa (2008), Turner and Tanzawa (1997) and Lee *et al*. (1999).

Since the discovery of endothelin (ET)-1 in 1988 (Yanagisawa *et al*., 1988; Inoue *et al*., 1989) the components of the ET signalling pathway have become established, comprising three structurally similar endogenous 21-amino acid peptides, ET-1, ET-2 and ET-3, that activate two GPCRs, ET_A_ (Arai *et al*., 1990) and ET_B_ (Sakurai *et al*., 1990). In humans, ET-2 differs from ET-1 by only two amino acids, whereas ET-3 differs by six amino acids representing more substantial changes. ET-3 is the only isoform that can distinguish between the two receptor subtypes, having a similar potency at the ET_A_ receptor as ET-1 and ET-2, but much lower affinity than these isoforms for the ET_B_ receptor (Figure 1). Structurally, ETs are unusual among the mammalian peptides in possessing two disulphide bridges. This feature is shared by the sarafotoxins, a family of peptides that were isolated from snake venom in the same year as the discovery of ET-1 (Takasaki *et al*., 1988), and that provided the first selective agonist at the ET_B_ receptor, sarafotoxin S6C (William *et al*., 1991).

A number of features of the ET signalling pathway are unusual compared with other peptidergic systems and these continue to intrigue investigators, with over a thousand ET-related papers still published each year. ET-1 is the most abundant isoform in the human cardiovascular system, predominantly released from endothelial cells to cause potent and unusually long-lasting vasoconstriction that may persist for many hours. ET-1 is a key mediator in regulating vascular function in the majority of organs systems balanced by opposing vasodilators, particularly NO, prostacyclin and endothelium-derived hyperpolarizing factor. Endothelial cell dysfunction occurs in pathophysiological conditions such as pulmonary arterial hypertension (PAH) and is associated with loss of these dilators and increased synthesis of ET. The consequence of this is vasoconstriction, proliferation of many different cell types particularly vascular smooth muscle, fibrosis and inflammation; processes associated with vascular remodelling. In disease, the deleterious actions of ET in the vasculature are mainly mediated by the ET_A_ receptor, whereas activation of ET_B_ receptors results in many of the beneficial effects of the peptide that frequently act as a regulatory counterbalance (Davenport and Maguire, 2006). The formation of the disulphide bridge in the ET peptides blocks the N-terminal amino acid conferring resistance to enzymic degradation in plasma. Internalization by ET_B_ scavenging receptors is therefore particularly important for termination of the ET signal in health and disease.

The major therapeutic strategy (Figure 1) to block the unwanted actions of ET in disease has been to use antagonists of ET_A_ receptors or both receptor subtypes (Palmer, 2009) with the first clinical application being bosentan in PAH (Rubin *et al*., 2002). More recently, a second strategy has started to be more widely tested in clinical trials using inhibitors of ET-converting enzymes 1 (ECE-1; Xu *et al*., 1994) and 2 (ECE-2; Emoto and Yanagisawa, 1995), the major biosynthetic pathway of ET (Figure 1) at least in the human vasculature (Russell and Davenport, 1999a,b). A third emerging strategy based on biosimilar agonists at the ET_B_ receptor (molecules similar, but not identical to the endogenous ligand) has led to the renaissance of IRL1620 as a clinical candidate in delivering anti-tumour drugs and in other pathophysiological conditions such as cerebral ischaemia.

The aim of this focused IUPHAR review is to highlight recent progress and some surprising new discoveries in the pharmacology of the ET system. The following references are recommended for more detailed information on specific ET research areas including the heart (Kelland and Webb, 2006; Kirkby *et al*., 2008; Kohan *et al*., 2012; Ohkita *et al*., 2012; Vignon-Zellweger *et al*., 2012; Drawnel *et al*., 2013), renal (Dhaun *et al*., 2011; 2012[Bibr b32],[Bibr b33]; Kohan *et al*., 2011a,b[Bibr b73],[Bibr b74]; Hyndman and Pollock, 2013), hypertension (Rautureau and Schiffrin, 2012; Speed and Pollock, 2013), PAH (Pernow *et al*., 2012; Rubin, 2012; Liu *et al*., 2013), cancer (Bagnato *et al*., 2011; Said and Theodorescu, 2012; Rosanò *et al*., 2013b), atherosclerosis and diabetes (Pernow *et al*., 2012).

## ET peptides

### Evidence for a new ET peptide: the ET-like domain peptide (ELDP)

The ELDP has been recently identified as a peptide corresponding to prepro-ET-1_93–166_ (Yuzgulen *et al*., 2013) immediately adjacent to the gene sequence encoding big ET-1. The 74-amino acid peptide has been detected by HPLC and specific double recognition site immunoassays in conditioned media from two cell lines, endothelial (EA.hy 926) and epithelial (A549), as well as from primary cell cultures of human aortic endothelial cells that are known to secrete ET-1. In the aortic endothelial cells, the peptide was co-synthesized and co-released with ET-1. Plasma levels in untreated patients were 6.5 pmol·L^−1^, which compares with typical basal levels of immunoreactive ET-1 of 5 pmol·L^−1^ (Davenport *et al*., 1990). Levels of ELDP were significantly elevated in patients with heart failure suggesting a potential use as a bio-marker. While no effect was observed on BP in the anaesthetized rat, intriguingly ELDP significantly increased the duration of the pressor response of ET-1 (0.3 nmol·kg^−1^, likely to be a submaximal dose). Pretreatment of rat mesenteric arteries with 10 nM ELDP also potentiated a submaximal response of ET-1 by fivefold (Yuzgulen *et al*., 2013). It is not unexpected that a second peptide sharing a cleavage site with ET-1 would also be co-released, but it is intriguing that the peptide was able to potentiate ET-1 responses *in vitro* and *in vivo.* It is not yet reported, using saturation or competition-binding experiments, whether ELDP binds directly to ET receptors, binds to an allosteric site or whether the peptide modulates ET responses by other mechanisms. Intriguingly, ELDP encompasses the sequence of the putative ‘endothelin-like peptide’ corresponding to prepro-ET-1_109–123_ proposed in the original *Nature* paper by Yanagisawa *et al*. (1988). Eight out of 15 residues in the corresponding sequence in ET-1 are identical and the four Cys residues are perfectly conserved and flanked by dibasic pairs that are recognized by endopeptidase processing enzymes, to yield a cleaved peptide. However, a synthetic peptide corresponding to this sequence was devoid of agonist or antagonist activity against ET-1, in vascular preparations (Cade *et al*., 1990).

### Global knockout of ET-2 gene reveals distinct phenotype compared with ET-1/ET_A_ and ET-3/ET_B_ gene deletions

ET–1-deficient homozygous mice die at birth of respiratory failure, which is secondary to severe craniofacial and cardiovascular abnormalities. ET_A_ receptor and ECE-1 knockout mice have similar morphological abnormalities (Kurihara *et al*., 1994; Clouthier *et al*., 1998; Yanagisawa *et al*., 1998). The phenotype is similar to a spectrum of human conditions, CATCH 22 (cardiac anomaly, abnormal face, thymic hypoplasia, cleft palate, hypocalcaemia, chromosome 22 deletions) and established the importance of the ET_A_/ET-1 signalling system for cardiovascular and craniofacial development. Gene deletions for ET-3 and ET_B_ receptors exhibit a different and non-overlapping phenotype to ET-1/ET_A_-deficient animals. They are viable at birth and survive for up to 8 weeks, but display aganglionic megacolon, as a result of absence of ganglion neurones, together with a disorder of the pigment in their coats (Baynash *et al*., 1994; Hosoda *et al*., 1994). In these mice, enteric nervous system precursors and neural crest-derived epidermal melanoblasts fail to colonize the intestine and skin. This phenotype resembles Hirschsprung's disease in man.

Deleting genes encoding all the key molecules, ET-1, ET-3, ET_A_, ET_B_, ECE-1 and ECE-2 has been accomplished in mice generating important information about their effect on phenotype. The deletion of the gene for ET-2 has now been reported. The physiological role of ET-2 has been puzzling. It had been assumed that the actions of ET-2 would be similar, if released, to the more widely distributed and abundant ET-1. Current antagonists block both ET-2 and ET-1 with the same potency and are not yet able to distinguish the actions of these peptides.

A key advance in the field was the generation by Chang *et al*. (2013) of a global ET-2 gene knockout mouse, which surprisingly exhibited a distinct phenotype to global ET-1 or ET-3 gene deletions. These mice showed severe growth retardation, internal starvation characterized by hypoglycaemia, ketonaemia and increased levels of starvation-induced genes. Mice were profoundly hypothermic and the median lifespan could be significantly extended by housing in a warm environment. The intestine was morphologically and functionally normal, which was unexpected as murine ET-2 (see Ling *et al*., 2013), also known as vasoactive intestinal contractor, is present throughout the gastrointestinal tract suggesting, in this tissue at least, in the absence of ET-2, ET-1 continues to mediate signalling. In agreement, intestinal epithelium-specific ET-2 knockout mice showed no abnormalities in growth and survival. In marked contrast, dramatic changes were observed in lung morphology and function. Mice had breathing difficulties after the first week exhibiting enlarged air spaces with substantial simplification of lung alveolar structure, larger lung capacities leading to abnormally elevated carbon dioxide (hypercapnia) and deficiency of oxygen (hypoxaemia) in the blood. Hypothermia and lung dysfunction might not be specific, but may be due to a secondary effect of internal starvation because of ET-2 deficiency. However, it is possible that these studies identify an important function for ET-2 in the pulmonary system. The authors showed that mRNA encoding ET-2 was only present in epithelial cells whereas receptor mRNA was mainly present in mesenchyme, consistent with a paracrine function for ET-2 in the lung.

### Pharmacological significance: is ET-2 the inducible isoform?

The dramatic effects on the lung suggest a crucial role for ET-2 at birth, at least in mice. The lungs, and potentially the heart, remain major therapeutic targets for ET antagonists in humans in the treatment of PAH. In rodents, ET-2 was less widely distributed than ET-1, mainly found in heart, lung, ovary, stomach and all regions of the intestine (de la Monte *et al*., 1995; Takizawa *et al*., 2005). ET-2 expression in human tissue was similar, being present in the human heart (Plumpton *et al*., 1996b), lung (Marciniak *et al*., 1992), kidney (Karet and Davenport, 1996), vasculature, (Howard *et al*., 1992) intestine and ovaries (Palanisamy *et al*., 2006), but has not been investigated in pathophysiological tissue. In humans, alternatively spliced mRNA variants encoding ET-2 have been detected with a specific pattern of distribution in various tissues. Some of these variants contain sites for the post-transcriptional processing of preproET-2 into mature ET-2, which may be altered in a tissue-specific manner (O'Reilly *et al*., 1993). The best established model of spatial and temporal ET-2 signalling is in the ovary, a highly vascular tissue, which undergoes cyclic changes as follicles grow, rupture and transform into corpora lutea and eggs are periodically released (Ko *et al*., 2012). In rats, low levels of ET-1 are constitutively expressed throughout the ovulatory cycle, whereas ET-2 is induced transiently at much higher concentrations during the period of ovulation to luteal phases (Ko *et al*., 2006). ET-2 is expressed in the granulosa cells of periovulatory follicles, but not during other stages of follicular development. In mice, induced superovulation results in a dramatic increase in ET-2 mRNA expression (Palanisamy *et al*., 2006). ET-2 expression surged in response to gonadotropin and quickly declined by 13 h, which coincided with the time of follicular rupture. Crucially, both ET receptor subtypes are present and their ratio does not seem to change. Thus, the ET-2 gene appears to be switched on only when increased levels of ET are required, with ET–2-mediated contraction being the final signal facilitating ovulation (Ling *et al*., 2013).

ET-1 signalling is well established in neural crest migration. In the developing mouse retina, constitutive over-expression of ET-2 affects vascular development by inhibiting endothelial cell migration across the retinal surface and subsequent endothelial cell invasion into the retina, an action mediated by ET_A_ receptors. Interestingly, over-expression is spatially localized as it has no obvious action on vascular structures in brain or skin (Rattner *et al*., 2013). Constitutive over-expression of ET-2 signalling also protected photoreceptors from light damage (Braunger *et al*., 2013). Similarly, Bramall *et al*. (2013) found expression of ET-2 mRNA was greatly increased in the photoreceptors of mouse models of inherited photoreceptor degeneration and, using the global ET-2 knockout mice, showed increased ET-2 expression was protective of the mutant photoreceptors.

### Case for re-evaluating the role of ET-2

Targeting the ET-2 gene in mice provides compelling evidence that, while both ET-1 and ET-2 can coexist in the same tissue compartments, there is a critical, but distinct ET-2 pathway. A key role has now been established for ET-2 in ovarian physiology. This may be accomplished at the level of gene expression, but differences may also exist in peptide synthesis by ECEs and chymase, which may allow the two ET peptide pathways to be distinguished pharmacologically and become separate drug targets. Additionally, pharmacological differences have been identified, for example ET-2 dissociates from receptors much more rapidly than ET-1 and higher affinity has been reported, for example in the brain (Ling *et al*., 2013). Detailed studies comparing rat mesenteric resistance and basilar arteries demonstrated that ET-1 and ET-2 initiate and maintain vasoconstriction by different downstream mechanisms raising the prospect of ‘biased signalling’ mediated by two structurally different agonists activating the same receptor (Compeer *et al*., 2013).

## Potential new therapeutic strategies exploiting ET_B_ receptor agonists

The pharmacological rationale for this strategy is that ET-1, tonically released from endothelial cells, also interacts with endothelial cell ET_B_ receptors. The importance of this counter-regulatory pathway has been underestimated to date. Endothelial cells line the vasculature of every organ and tissue in the body that receives blood supply. Although the cells represent ∼1% of the weight of the vessel wall, they have a combined mass comparable with some endocrine glands. Crucially, ET-1 feeding back onto endothelial receptors to release NO not only limits ET_A_-mediated vasoconstriction by stimulation of vascular cGMP, but also limits further ET-1 release. Thus in the vasculature, NO and other dilators are crucial in balancing the ET system, but these may be reduced or absent in pathophysiological conditions.

ET-1^+/−^ heterozygous mice developed *elevated* BP and mild hypertension, rather than the fall in BP that might have been expected. Partial deletion of the gene allows survival and produced lower levels of ET-1 in plasma and lung tissue than wild type (Kurihara *et al*., 1994). These results suggest that ET-1 has an essential physiological role in cardiovascular homeostasis. Low levels promote vasodilatation whereas higher and pathophysiological concentrations of ET-1 increase BP and total peripheral vascular resistance. While ET_A_ receptor selective antagonists such as BQ123 (Ihara *et al*., 1992) cause the expected vasodilatation in humans (Haynes and Webb, 1994), the ET_B_ receptor selective antagonist BQ788 (Ishikawa *et al*., 1994) caused systemic *vasoconstriction* in healthy volunteers, showing that the main consequence of activation of endothelial ET_B_ receptors by tonically secreted ET-1 was the physiological basal release of NO (Love *et al*., 2000). In agreement, initial vasodilatation can be detected in the human forearm vascular bed following infusion of low concentrations of ET-1 whereas higher doses caused sustained vasoconstriction (Kiowski *et al*., 1991). A contribution to vasoconstriction may also be the result of occupancy by ET-1 of the clearance ET_B_ receptors causing an ET_A_-mediated vasoconstriction.

### ET_B_ agonists in chemotherapy: IRL1620

ET-1 acting on ET_A_ receptors has been proposed to stimulate cell proliferation, migration, invasion, osteogenesis and angiogenesis in several cancers. New vessels forming in tumours are characterized by high densities of ET_A_ receptors in smooth muscle, for example in glioblastoma multiforme in the brain (Harland *et al*., 1998). Conversely, ET_B_ receptors may oppose tumour progression by promoting apoptosis and clearing ET-1 (Bagnato *et al*., 2011; Rosanò *et al*., 2013b). The strategy of stimulating ET_B_ receptors to cause transient vasodilatation is being developed to increase the penetration of cytotoxic anti-tumour agents into tumours and to minimize the concentration in healthy tissue.

IRL1620 was originally developed as a tool compound (Takai *et al*., 1992). The N-terminus has an N-succinyl modification, which is likely to reduce metabolism by non-specific peptidases, but it is not orally active and requires injection. Despite these unpromising pharmacokinetic features, it is being used *in vivo* and has emerged as a possible clinical candidate in improving the delivery of drugs to tumours. IRL1620 infused into rats improved the efficacy of doxorubicin and 5-flurouracil by significantly increasing the amount of drug in tumours in rat models of prostrate and breast cancer. In addition, radiation-induced reduction in tumour volume was enhanced, suggesting IRL1620 can significantly increase the efficacy of radiotherapy in the treatment of solid tumours. The results suggest that for a given dose of drug, the efficacy in reducing the tumour could be improved (Gulati and Rai, 2004; Rajeshkumar *et al*., 2005a,b[Bibr b121],[Bibr b122]; Lenaz *et al*., 2006; Rai *et al*., 2006; Gulati *et al*., 2012). A phase I trial to determine the safety, tolerability, pharmacokinetics and pharmacodynamics of IRL1620 (known as SPI-1620 licensed by Spectrum Pharmaceuticals, Technology Drive Irvine, CA, USA) in patients with recurrent progressive carcinoma has been successfully completed and shown to selectively and transiently increase tumour blood flow (Gulati *et al*., 2012; http://www.cancer.gov/clinicaltrials). A phase II trial was initiated in 2013 to determine the effectiveness of SPI-1620 in combination with docetaxel in patients with advanced biliary cancer (http://clinicaltrials.gov/ct2/show/NCT01773785) and in combination with docetaxel compared with docetaxel alone for patients with non small-cell lung cancer after failure of platinum-based chemotherapy (http://clinicaltrials.gov/show/NCT01741155).

### ET_B_ agonists in neuroprotection

The human brain contains the highest density of ET receptors, with the ET_B_ receptor subtype comprising about 90%, in areas such as cerebral cortex (Harland *et al*., 1998). Binding and functional studies have demonstrated glia mainly express ET_B_ receptors whereas ET_A_ receptors are localized mainly on neurones (Morton and Davenport, 1992). Smooth muscle cells from large arteries and small intracerebral vessels only express ET_A_ receptors (Adner *et al*., 1994; Harland *et al*., 1995; Pierre and Davenport, 1999) with endothelial cell ET_B_ receptors mediating relaxation (Lucas *et al*., 1996). The small pial arteries and arterioles penetrating into the brain play a major role in the maintenance of cerebral blood flow (auto-regulation). These vessels are particularly sensitive to ET-1 compared with peripheral vessels and the peptide has been a long-standing candidate in the genesis or maintenance of cerebrovascular disorders such as delayed vasospasm associated with subarachnoid haemorrhage or stroke. ET-1 does not cross the blood–brain barrier from the plasma, but may do so when compromised by subarachnoid haemorrhage, stroke or head injury. Strategies for targeting cerebrovascular disease have focused previously on the use of ET receptor antagonists, firstly to block vascular receptors mediating cerebrovasospasm that may be responsible for delayed cerebral ischaemia seen after aneurysmal subarachnoid haemorrhage and could contribute to ischaemic core volume in stroke. Secondly, to block neural receptors that mediate increases in intracellular free calcium (Morton and Davenport, 1992) and initiate the pathophysiological processes leading to neuronal death.

A new emerging strategy is to use ET_B_ receptor agonists such as IRL1620 to provide vasodilatation and neuroprotection. The peptide reduced neurological damage following permanent middle cerebral artery occlusion in rats, a model of focal ischaemic stroke. Animals received i.v. injections of IRL1620 after the occlusion, which dramatically reduced infarct volume (by more than 80% in the acute and 70% in the chronic study), prevented cerebral oedema, reduced oxidative stress markers and improved all neurological and motor function for up to 7 days (Leonard *et al*., 2011; 2012[Bibr b82],[Bibr b83]; Leonard and Gulati, 2013). Rats treated with the amyloid peptide Aβ_1–40_ administered into the intracerebral vessels display increased markers of oxidative stress in the brain. IRL1620 significantly reduced oxidative stress and importantly the cognitive impairment (Briyal *et al*., 2014). As discussed later, a reduction in ECE activity is associated with accumulation of amyloid β-peptide and neurotoxicity early in progression of Alzheimer's disease (Eckman *et al*., 2001; 2003; 2006[Bibr b39],[Bibr b40],[Bibr b41]; Pacheco-Quinto and Eckman, 2013). These results are limited to disease models in a single species, and it is unclear whether the molecular mechanisms would translate to humans, but taken together, they suggest that an ET_B_ receptor agonist might offer a new therapeutic strategy in Alzheimer's disease and provide neuroprotection following cerebral ischaemia in conditions such as stroke.

### No evidence for further ET_B_ receptor subtypes

Previous studies have suggested that ET_B_ receptors could be further subdivided into ET_B1_ present on endothelial cells and ET_B2_ on smooth muscle cells. Studies continue to be published with this misleading nomenclature, but current evidence only supports the existence of two subtypes, ET_A_ and ET_B_, according to NC-IUPHAR nomenclature (Davenport, 2002; Alexander *et al*., 2013a,b[Bibr b3],[Bibr b4]). Firstly, Mizuguchi *et al*. (1997) demonstrated unequivocally that in ET_B_ receptor knockout mice, both the direct constrictor and indirect vasodilator responses to the ET_B_ agonist sarafotoxin S6C were abolished. Selective deletion of endothelial ET_B_ receptors in mice (demonstrated by autoradiography to leave unaltered ET_B_ receptors expressed by other cell types) impaired, as expected, the clearance of an i.v. bolus of labelled ET-1 compared with controls (Bagnall *et al*., 2006; Kelland *et al*., 2010). Secondly, Flynn *et al*. (1998) were unable to distinguish pharmacologically, in extensive competition-binding experiments, between ET_B_ receptors expressed by human isolated endothelial and smooth muscle cells in culture. In concordance, saturation-binding assays in human tissue always found ET_B_ radiolabelled ligands bound with a single affinity and Hill slopes close to unity with no suggestion of further subtypes (Molenaar *et al*., 1992; 1993[Bibr b99],[Bibr b100]; Nambi *et al*., 1994) or in competition-binding versus radiolabelled ET-1 in human native (Peter and Davenport, 1995; 1996[Bibr b114],[Bibr b115]; Russell and Davenport, 1996) or recombinant ET_B_ receptors (Nambi *et al*., 1994; Reynolds *et al*., 1995). Clozel and Gray (1995) showed that endothelial and smooth muscle ET_B_ receptors cannot be distinguished functionally.

### Do ETs interact with any other GPCRs?

The virtually complete sequencing of the human genome has allowed the identification of all of the human gene sequences that could potentially encode GPCRs that are currently classified as ‘orphan’ to indicate that their endogenous ligand is not yet known (Foord *et al*., 2005; Davenport *et al*., 2013). In this catalogue, the most closely related to the ET_A_ and ET_B_ receptor subtypes are the orphan receptors GPR37 (also know as ET receptor type B-like or Parkin-associated ET receptor-like receptor) and its related receptor, GPR37L1. A recent high-throughput screen tested ∼10 000 biologically active compounds for binding to 82 remaining orphan GPCRs. None of the ∼20 ET peptides tested at high concentration (including all three mature isoform and their corresponding big ET precursors, C-terminal metabolites, BQ123 and the ET_B_ receptor agonist BQ3020) activated any of the expressed receptors, including GPR37 or GPR37L, supporting the established concept of ETs binding to only two receptor subtypes. Two orphan neuropeptides, prosaptide and prosaposin, have recently been proposed as cognate ligands for GPR37 and GPR37L (Meyer *et al*., 2013).

## Clinical application of ET antagonists

### Bosentan, ambrisentan and withdrawal of sitaxentan

PAH is a progressive condition with no cure and has a major impact on the ability to lead a normal life. It is an orphan disease (∼100 000 patients in US and Europe). PAH involves constriction of pulmonary arteries and is characterized by high BP in the lungs, ultimately leading to right heart failure and death. A number of pathways have been implicated in the development of PAH including bone morphogenetic proteins, prostacyclin and ET-1. Restoring the imbalance between constriction and vasodilatation of blood vessels is the basis for current medical therapies, although the cause of death is right heart failure. Although ET_A_ receptors are significantly increased in the right ventricle of patients with PAH (Kuc *et al*., 2014) and in the left ventricle of patients with heart failure (Zolk *et al*., 1999), surprisingly, ET receptor antagonists have clinical efficacy in the former, but not the latter group (Kohan *et al*., 2012).

**Figure 2:**
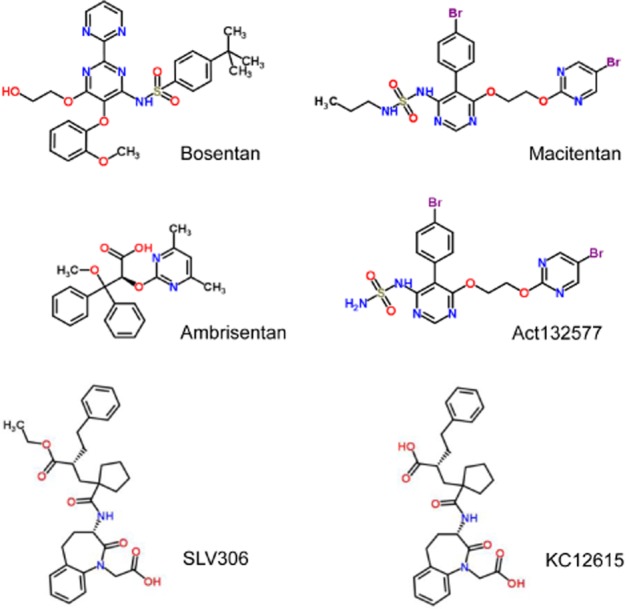
Structures of ET receptor antagonists in clinical use bosentan, ambrisentan and macitentan. The structures of the NEP/ECE inhibitor pro-drug SLV306 and its active metabolite are also shown.

**Figure 3:**
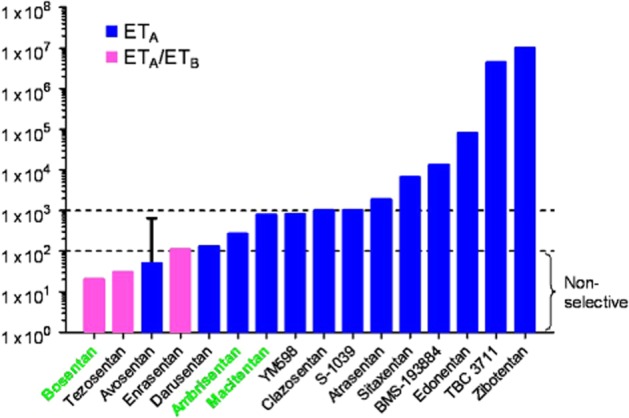
Selectivity of ET receptor antagonists for ET_A_ versus ET_B_ receptors shown on the vertical axis as reported by the companies that discovered the compounds. Selectivity was mainly determined by measuring affinity constants in separate competition assays against [^125^I]-ET-1 using human recombinant ET_A_ versus ET_B_ receptors and may not reflect selectivity measured in clinically relevant native tissues. Bosentan, ambrisentan and macitentan are currently approved for clinical use and are highlighted.

Bosentan (Tracleer, Ro47-0203) was the first ET receptor antagonist to be introduced into the clinic for the treatment of PAH (Rubin *et al*., 2002) and, as an orally acting agent, at the time represented a major advance over existing therapies such as prostacyclin analogues. Bosentan is classified as a mixed ET_A_/ET_B_ receptor antagonist blocking both receptors (Figures 2 and 3). The second antagonist to enter the clinic in 2007 was ambrisentan (Letairis, Volibris, LU208075, Figures 2 and 3), which was reported to display some ET_A_ receptor selectivity (Vatter and Seifert, 2006) followed by the most highly selective ET_A_ receptor antagonist sitaxentan (Thelin, TBC11251) (Barst *et al*., 2004). While hepatotoxicity is a known side effect of ET antagonists, it is usually reversible and related to dose. Unfortunately, cases of idiosyncratic hepatitis resulting in acute liver failure leading to death have been reported with sitaxentan and the compound was withdrawn in 2010 (Don *et al*., 2012).

### Next generation of ET antagonists: macitentan

Despite the current use of ET receptor antagonists and drugs targeting the two other principal pathways- that of NO with PDE5 inhibitors and that involving prostacyclin (PGI_2_) – meta-analysis of PAH trials shows existing therapies only moderately increased the most widely used objective evaluation of functional exercise capacity (6 min walk distance) by 11%. The prognosis for patients with PAH remains poor with ∼15% mortality within 1 year. There remains an urgent need for new efficacious treatments that has lead to the development of macitentan.

Macitentan (Opsumit, ACT-064992, Figures 2 and 3) represents the next generation of orally active ET receptor antagonists and was developed by modifying the structure of bosentan to improve efficacy and tolerability (Bolli *et al*., 2012). Macitentan is described as a dual antagonist that blocks both ET_A_ and ET_B_ receptors and it inhibited [^125^I]-ET-1 binding to human recombinant ET_A_ receptors with an IC_50_ of 0.2nM and to ET_B_ receptors with an IC_50_ of 391 nM. On the basis of these results, macitentan displays about 800-fold selectivity. A phase III clinical trial was successfully completed in 2012 (Pulido *et al*., 2013), and the compound gained approval from the US FDA in 2013 for the treatment of PAH. Macitentan is metabolized by the cytochrome P450 system, predominantly CYP3A4 and to a lesser extent the CYP2C19 iso-enzyme. Unlike other antagonists currently in use, one of the metabolites of macitentan, ACT-132577 (Figure 2), is pharmacologically active. Although it has a lower potency than the parent compound ACT-132577 reaches higher plasma concentrations, with a longer half-life of about 48 h (Iglarz *et al*., 2008; Sidharta *et al*., 2011; 2013a, b). These factors are likely to contribute to improved activity of macitentan compared with bosentan. While *in vitro* studies suggested macitentan was likely to interact with other drugs (Weiss *et al*., 2013), other observed pharmacokinetic benefits included fewer interactions with other drugs at clinically used concentrations, no requirement to alter doses in patients with renal or hepatic impairment, improved hepatic safety and reduced oedema/fluid retention compared with bosentan. Key differences were also identified in the pharmacodynamic parameters. For example, in calcium release assays macitentan was more potent (*K*_B_ = 0.1 nM) than bosentan (*K*_B_ = 1.1 nM) and had a significantly longer receptor occupancy (17 min compared with 70 s) (Iglarz *et al*., 2008; Bruderer *et al*., 2012a,b; 2013; Gatfield *et al*., 2012). The authors suggested that the macitentan binding site differed slightly from the bosentan binding site and that this difference in interaction with amino acids in the receptor contributed to the slow dissociation of macitentan from the receptor, particularly leading to insurmountable antagonism. A number of clinical trials are actively recruiting (Patel and McKeage, 2014) including the use of macitentan for the treatment of digital ulcers in patients with systemic sclerosis, Eisenmenger's syndrome and, perhaps the most challenging, in patients with brain tumours (glioblastoma).

## Compounds interacting with ET-1 synthesis and metabolism

Members of the neprilysin (NEP)-like family of zinc metalloendopeptidases play key roles in the ET pathway (Turner and Murphy, 1996; Turner *et al*., 2001). Neutral endopeptidase (NEP) is a membrane-bound thermolysin-like zinc metalloendopeptidase, which is particularly abundant in human kidneys. The enzyme metabolizes a number of peptides including enkephalins, tachykinins, natriuretic peptides as well as the ETs (Turner and Tanzawa, 1997). Inactivation of ET-1 is via a two-stage process, opening of the Ser^5^–Leu^6^ bond, followed by cleavage at the amino side of Ile^19^ resulting in an inactive peptide, which is inhibited by phosphoramidon (Skolovsky *et al*., 1990). Pharmacological intervention in the pathway is challenging because NEP-like enzymes also include the synthetic enzymes ECE-1, ECE-2 and KELL. The ECEs are also inhibited by phosphoramidon and ECE inhibitors currently in clinical trials have significant NEP inhibitory activity and it seems counter-intuitive to inhibit the degradative pathway. However, in practice, inactivation of ET-1 is thought to be mainly via binding and internalization of the ET_B_ receptor and ET-1 is essentially stable in plasma. Binding to ET_B_ receptors, particularly in those organs such as the lung expressing high densities of the subtype, are critical for inactivation of the peptide. After internalization of the ligand–receptor complex to the lysosome, ET-1 is thought to be degraded, like other peptides, by cathepsin A. In support, cathepsin A knockout mice showed reduced ET-1 degradation and significantly increased arterial BP. In humans, genetic defects of cathepsin A include hypertension and cardiomyopathies (Seyrantepe *et al*., 2008).

### ECE-1

It is now well established that ET is synthesized in a three-step process, with pre-pro-ET-1 initially cleaved by a signal peptidase to proET-1, which is in turn cleaved by a furin enzyme to an inactive precursor big ET-1 (Figure 1). Although low MW inhibitors of furins have been reported, furins cleave a number of other proteins to mature or active forms and therefore are not an easy tractable drug target for selectively reducing ET-1, without altering other pathways. Targeting the ECE enzymes responsible for transformation of big ET-1 to the mature, biologically active ET-1 has been more promising (Xu *et al*., 1994; Turner and Murphy, 1996). In humans there are four isoforms, ECE-1a–d, derived from a single gene by the action of different promoters. Structurally, they differ only in the amino acid sequence of the extreme N-terminus. ECE-1 localizes to the small secretory vesicles of the constitutive pathway from where ET-1 is continuously released to maintain normal vascular tone. Unusually for vasoactive peptides, ET-1 is also synthesized by ECE-1 and stored in specialized Weibel–Palade bodies within endothelial cells until its release following an external physiological or pathophysiological stimulus (the regulated pathway) to produce further vasoconstriction (Russell *et al*., 1998a,b; Russell and Davenport, 1999b).

In addition to intracellular endothelial cell ECE, the enzyme is also present on vascular smooth muscle, efficiently converts big ET-1 in human vessels *in vitro* and is up-regulated in atherosclerosis (Maguire *et al*., 1997; Maguire and Davenport, 1998). Given the larger volume of the smooth muscle compared with the single layer of endothelium, smooth muscle ECE may be a more important source of ET-1 in pathophysiological conditions.

### ECE-2

ET-1 is also synthesized by a second membrane-bound metalloprotease, ECE-2 (Emoto and Yanagisawa, 1995; Yanagisawa *et al*., 2000; Lorenzo *et al*., 2001) with ∼60% sequence similarity to ECE-1. It is distinguishable from ECE-1 by having an optimum pH of 5.5 for activity. In human endothelial cells, ECE-2 was localized to the acidified environment of vesicles of the secretory pathway, but unlike ECE-1 it is not found in storage granules (Russell and Davenport, 1999b). Four isoforms exist, differing in their N-terminus: ECE-2a-1 and ECE-2a-2 are expressed predominantly in peripheral tissues and ECE-2b-1 and ECE-2b-2 in the brain, possibly representing the neuronal isoforms (Ikeda *et al*., 2002). The physiological importance of this pathway for ET-1 synthesis remains to be determined, as ECE-2 also metabolizes other peptides such as bradykinin. However, the requirement for an acidic pH suggests a role in pathophysiological conditions associated with low pH such as ischaemia. ECE-1/ECE-2 knockout mice display increased developmental defects compared with deletion of ECE-1 or ECE-2.

### Alternative, non-ECE synthetic pathway: chymase

ET-1 can also be synthesized indirectly by chymase, a serine protease present in mast cells. Big ET-1 is converted to ET-1_1–31_ by cleaving the Tyr^31^–Gly^32^ bond (Figure 1), which in turn is converted to the mature peptide via Trp^21^–Val^22^ bond (Fecteau *et al*., 2005; D'Orleans-Juste *et al*., 2008). The existence of an alternative pathway was originally predicted when ET-1 and ET-2 were detected in embryos of the ECE-1/ECE-2 double-knockout mouse (Yanagisawa *et al*., 2000).

The importance of this alternative pathway remains unclear, but importantly ET-1_1–31_ was equipotent with big ET-1 in causing vasoconstriction in human isolated vessels, including coronary arteries, and this was associated with the appearance of measurable levels of ET-1 in the bathing medium. ET-1_1–31_ displayed no selectivity between ET_A_ and ET_B_ receptors in human heart and vasoconstriction was fully blocked by ET_A_ receptor selective antagonists, reflecting the predominance of the ET_A_ receptor on vascular smooth muscle (Maguire *et al*., 2001; Maguire and Davenport, 2004). The precise physiological role of mast cells within human blood vessels is unclear, but following degranulation, which may occur under pathophysiological conditions, the mast cell chymase is associated with interstitial spaces with the potential to convert circulating big ET-1 and provide a further source of ET-1. Mast cell expression is increased in cardiovascular disease, for example in atherosclerotic lesions. It is therefore possible that the contribution of this pathway within the vasculature, leading to over-expression of ET-1, may be underestimated particularly in conditions of endothelial malfunction where opposing levels of endogenous vasodilators may be reduced. It is unclear whether under conditions of NEP/ECE inhibition the rising levels of big ET-1 would favour increased conversion by the serine protease pathway, thus increasing the pressor effect via ET_A_ receptors or whether excretion of unmetabolized big ET-1 by the kidney would be sufficient to remove the elevated levels of precursors (Johnström *et al*., 2010).

### KELL and ET-3 synthesis

Although big ET-3 is converted by ECE-1 to ET-3, owing to difference in the C-terminus the efficiency is much less than for ET-1. In contrast, big ET-3 is reported to be efficiently converted by KELL (Lee *et al*., 1999). KELL is a membrane-bound glycoprotein expressed in human erythrocytes and one of the major antigens; it is also related to mammalian NEP-like enzymes including ECE-1 and ECE-2 (Turner and Tanzawa, 1997). If KELL is the main synthetic pathway for ET-3, a possible benefit of inhibiting ECE would be to increase the ratio of ET-3 to ET-1, which could then differentially produce beneficial vasodilatation via the ET_B_ receptor, but this speculative hypothesis has not been tested.

### Pharmacological inhibition of ECE by research compounds

A combination of phosphoramidon and thiorphan has been widely used to identify ECE activity. This is based on the finding that the conversion of big ET-1 to ET-1 is inhibited by phosphoramidon, but not by thiorphan, and has been shown both *in vitro* and *in vivo*. Importantly for evaluating the significance of animal models, both compounds have also been used in clinical studies to characterize big ET-1 conversion (see Webb, 1995; Plumpton *et al*., 1996a; Hand *et al*., 1999). Low MW, non-peptide ECE inhibitors have been developed and one that has been widely used *in vitro* and *in vivo* animal models and is commercially available is CGS26303 (De Lombaert *et al*., 1994). CGS26303 inhibited conversion of all three big ETs in human isolated blood vessels but, importantly, did not interfere with the interaction of mature peptides with ET receptors (Yap *et al*., 2000). Although primarily an NEP inhibitor, SOL1, a more recent combined NEP/ECE non-peptide inhibitor with modest inhibition of ECE-1 *in vitro*, was remarkably potent *in vivo*, fully blocking the big ET–1-induced rise in BP at a dose of 10 μmol·kg^−1^ (Nelissen *et al*., 2012).

A disadvantage of using phosphoramidon is that it is not selective for ECE. An alternative tool compound is PD159790, which inhibits ECE-1 with an IC_50_ value of 3 μM; at this concentration the compound is selective for ECE-1 over NEP (Ahn *et al*., 1998). PD159790 has been shown experimentally in HUVECs to inhibit conversion of big ET-1 at pH 6.9, optimum for ECE-1, but did not affect big ET-1 conversion to the mature peptide at pH 5.4, optimum for ECE-2 (Russell and Davenport, 1999a). The compound did not inhibit the further metabolism of ET-1_1–31_, the chymase product of big ET-1 (Maguire *et al*., 2001) and can be used to distinguish between the three different pathways for ET synthesis. While the mature peptide is located in intracellular Weibel–Palade bodies or secretory vesicles within endothelial cells and a proportion of big ET-1 is converted to ET-1 intracellularly, it is not reported whether ECE inhibitors can cross the plasma membrane to access these intracellular sites. The main effects of these inhibitors may be on external ECE. In agreement with this proposal, the SLV306 metabolite KC-12615 (see later) effectively prevented conversion of exogenous big ET-1 in human vasculature (Seed *et al*., 2012).

### Emerging NEP/ECE inhibitors

Selective inhibitors of ECE have not progressed into clinical applications. SLV306 (daglutril, Figure 2) is an orally active, mixed enzyme inhibitor of both ECE and NEP. It is a pro-drug being converted *in vivo* to the active metabolite, KC-12615. This latter molecule has a pharmacological profile similar to phosphoramidon, inhibiting NEP in the nanomolar range, but with more modest inhibition in the micromolar range for ECE (Meil *et al*., 1998; Jeng *et al*., 2002). The therapeutic basis is that while inhibition of NEP alone increased plasma concentrations of atrial natriuretic factor (ANP) to cause vasodilatation, NEP inhibitors are ineffective as anti-hypertensives, probably because NEP also degrades vasoconstrictor peptides including ET. A combined ECE/NEP inhibitor would be predicted to reduce the systemic conversion of big ET-1 to the mature peptide and increase dilator peptides such as ANP. SLV306 is well tolerated with few or none of the side effects such as increases in liver function, oedema, observed with ET receptor antagonists (Dickstein *et al*., 2004; Parvanova *et al*., 2013). A potential disadvantage is big ET-1 might still be converted to ET-1 by an alternative pathway such as chymase. However, in animal models with normal renal function, this did not occur and big ET-1 labelled with the positron emitter ^18^F was rapidly accumulated unchanged in the kidney following inhibition of NEP/ECE, with no evidence of conversion by another pathway (Johnström *et al*., 2010).

The effect of a combined NEP/ECE inhibitor has been tested in volunteers in a randomized, double-blind trial. Following oral administration of three increasing doses of SLV306 (to reach an average target concentration of 75, 300, 1200 ng·mL^−1^ of the active metabolite KC-12615), big ET-1 was infused into 13 male volunteers at a rate of 8 and 12 pmol·kg^−1^·min^−1^ (20 min each). At the two highest concentrations tested, SLV306 dose-dependently attenuated the rise in BP after big ET-1 infusion. There was a significant increase in circulating big ET-1 levels compared with placebo, indicating that SLV306 was inhibiting an increasing proportion of endogenous ECE activity. Importantly, plasma ANP concentrations also significantly increased, consistent with systemic NEP inhibition (Seed *et al*., 2012).

### SLV306 in animal models and patients with type 2 diabetes and nephropathy

Diabetes causes activation of the renal ET system, which leads to progressive renal damage by cell proliferation and interstitial inflammation. Inhibitors of the renin–angiotensin system are widely used in treatment for hypertensive patients with type 2 diabetes, but are less effective in the advanced stages of diabetic renal disease. Studies in an animal model suggested that SLV306 had a similar efficacy to the angiotensin converting enzyme (ACE) inhibitor captopril in reducing proteinuria and preventing nephrosclerosis (Thone-Reinke *et al*., 2004). In this study, rats were treated with streptozotocin for twenty weeks and the effects of SLV306 (30 mg·kg^−1^ per day) compared with those of captopril (10 mg·kg^−1^ per day). SLV306 significantly decreased renal interstitial matrix content as well as protein and albumin excretion in diabetic rats, independent of BP and was as effective as captopril. These results suggested SLV306 treatment on top of blocking the renin–angiotensin system might have an additional benefit in reducing BP and improving renal function.

Parvanova *et al*. (2013) tested the efficacy of SLV306 in 45 patients with type 2 diabetes mellitus who had albuminuria and were already receiving the angiotensin receptor antagonist losartan, together with up to two additional anti-hypertensive drugs, in a randomized, crossover, double-blind, placebo-controlled trial. Although 8 weeks of treatment with SLV306 together with losartan did not significantly alter urinary albumin excretion or renal haemodynamic measures, the authors showed for the first time that the combination decreased ambulatory BP (particularly for systolic hypertension) in this patient group that are often resistant to treatment. There was a small, but significant increase in plasma big ET-1, consistent with ECE inhibition, but surprisingly not in pro-ANP. Increases in the natriuretic peptides was measured in healthy volunteers by Seed *et al*. (2012). Interestingly, the effect of SLV306 in this study on BP was higher at night (10 versus 12 mmHg). This is of potential importance as increased hypertension at night is a strong cardiovascular risk factor in this patient population. The molecular mechanism is not yet known, as plasma levels of big ET-1 were not reported separately for daytime versus night. The study was comparatively short and did not reveal significant changes in albumin excretion as predicted from animal studies. Long-term trials are required to determine whether the observed lowering of BP by SLV306 will translate into longer-term renal and cardio-protection.

### SLV306 and congestive heart failure

The effect of three single oral doses of SLV306 was tested in patients with congestive heart failure who underwent right-sided heart catheterization in a randomized, double-blind, placebo-controlled design (Dickstein *et al*., 2004). Pulmonary pressures and right atrial pressure decreased significantly in all SLV306 dose groups with the maximum decrease occurring at 6–8 h. Despite plasma levels of the drug increasing with dose, there was no clear dose–response relationship, which may have been the result of the comparatively small numbers (18–20) in the study.

### Insight in NEP/ECE inhibition from animal models

The efficacy of inhibiting NEP/ECE in animal models associated with increases in the ET signalling pathway has provided clues to future clinical applications. The development of nephropathy in diabetes is associated with a poor outcome, eventually leading to end-stage renal disease. In patients with diabetes, urinary excretion of protein and albumin rises and is associated with increased risk of cardiovascular disease. In diabetic rats, SLV306 decreased renal matrix protein content, protein and albumin excretion. The magnitude of these effects was comparable to those of ACE inhibition and independent of BP (Thone-Reinke *et al*., 2004). Currently, there are few drugs for the treatment of chronic renal failure. SLV338, a NEP/ECE inhibitor, abolished renal tissue damage (interstitial fibrosis, glomerulosclerosis, renal arterial remodelling) in rat models of both acute kidney failure as well as chronic renal failure. The compound preserved kidney function and reduced mortality (Sharkovska *et al*., 2011). In spontaneously hypertensive stroke-prone rats, SLV338 significantly improved survival in comparison with the vehicle-treated group in a BP-independent manner and could offer a new therapeutic approach for primary stroke prevention and improvement of mortality (Wengenmayer *et al*., 2011). SLV338 was also tested for cardiac protection in rat model of experimental renovascular hypertension (two-kidney, one-clip). SLV338 prevented cardiac remodelling to the same extent as losartan, but in a BP-independent manner. This effect was at least partly mediated via suppression of cardiac TGF-β1 expression (Kalk *et al*., 2011).

ET has been proposed to be a mediator in toxic liver injury. However, while SLV338 largely prevented the activation of the ET system it did not prevent D-galactosamine-induced acute liver injury in rats. The authors speculated that SLV388 should be tested in a less severe model of liver injury, as very severe intoxication might not be relevantly amenable to pharmacological interventions (Hocher *et al*., 2011).

### ECE-1 and amyloid deposition

The strategy in the cardiovascular and renal systems has been to *inhibit* ECE-1 activity. However, evidence is emerging that ECE-1 may function in the brain as a novel enzyme degrading amyloid β-peptides at several sites. Deposition of amyloid in the brain in Alzheimer's disease is determined not only by its production, but also by its catabolism. ECE-1 inhibition produces, in addition to extracellular accumulation, accumulation of intracellular amyloid β-peptides within endosomal/lysosomal and autophagic vesicles and an intracellular pool, is partly regulated by ECE activity at the sites of production. Reduction in ECE activity leads to accumulation of amyloid β-peptide, which is associated with neurotoxicity early in progression of Alzheimer's disease (Eckman *et al*., 2001; 2003; 2006[Bibr b39],[Bibr b40],[Bibr b41]; Pacheco-Quinto and Eckman, 2013). The clearance of Aβ_1–40_ in mice was almost completely inhibited by phosphoramidon as well as insulin indicating that human Aβ_1–40_ was degraded, at least in part, by a phosphoramidon-sensitive pathway, implicating both ECE and NEP (Ito *et al*., 2013).

To date, these investigations have comprised *in vitro* or *in vivo* rodent studies It is not yet clear whether *enhancing* ECE-1 activity is a potential drug target in Alzheimer's disease rather than inhibiting ECE-1, as in the periphery. ECE-like immunoreactivity has been localized to afferent and efferent fibres of neurones and neuronal cell bodies of mixed morphology in human brain (Giaid *et al*., 1991). Drugs increasing ECE activity such as enzyme enhancers or recombinant ECE would have to cross the blood–brain barrier, and it is not clear what effect this would have on ET signalling in the periphery.

## What are the new ET drug targets in the future?

### Epigentics

Epigenetics can be defined as heritable changes in phenotype through mechanisms other than changes in DNA sequence. Epigenetic changes will therefore be preserved when cells divide and affect normal development and disease progression. Processes mediating epigenetic regulation include DNA methylation and histone modification, which involves post-translational covalent modification of histone proteins by a range of writers, erasers and readers. This in turn modulates the ability of associated DNA to be transcribed. The histone code is read by specific families of proteins such as the bromodomains. These are of pharmacological significance because of the recent discovery of low MW inhibitors, which selectively modulate gene expression (Prinjha *et al*., 2012).

Epigenetic regulation is of particular importance in the ET pathway with transcription being the primary level of ET-1 regulation of the gene *EDN1* by histone modifications and DNA methylation (Welch *et al*., 2013). Silencing of the *EDNRB* gene by DNA methylation during development of tumours results in the down-regulation of the receptor. As a result, promotion of apoptosis via the ET_B_ receptor is reduced or lost, suggesting the ET_B_ receptor could be a target for epigenetic drugs or ET_B_ agonists where ET may be the cause of some tumour types, including melanomas and oligodendrogliomas (Bagnato *et al*., 2011). Intriguingly, epigenetic inactivation of ET-2 and ET-3 mRNA and protein was found in rat and human colon tumours and cancer cell lines, as a result of hypermethylation of *EDN2* and *EDN3* genes. Restoring expression of ET-2 and ET-3 in human cells significantly attenuated the migration and invasion of human colon cancer cells (Wang *et al*., 2013). As ET-3 displays high affinity for the ET_B_ receptor, forced expression of ET-3 might antagonize the actions of ET-1 mediated through ET_A_ receptors. Such a mechanism would be consistent with proposed beneficial effects of the ET_B_ receptor agonist, IRL-1620, in cancer.

### Life before birth – is ET a critical pathway?

Maternal malnutrition and uteroplacental vascular insufficiency causes foetal growth restriction or intrauterine growth retardation. Low birthweight is linked to the later development of cardiovascular disease and hypertension. Maternal treatment with dexamethasone increased ET-1 constrictor responses and ET_A_ receptor expression in placental arteries from the foetus (Docherty *et al*., 2001; Kutzler *et al*., 2003). Maternal nutrient restriction increased the histone acetylation and hypoxia inducible factor-1α (HIF-1α) binding levels in the ET-1 gene promoter of pulmonary vein endothelial cells (PVECs) in intrauterine growth restriction (IUGR) newborn rats, and continued up to 6 weeks after birth. These epigenetic changes could result in an IUGR rat being highly sensitive to hypoxia later in life, causing more significant PAH or pulmonary vascular remodelling. Recently, Xu *et al*. (2013) have shown that restricting the diets of pregnant rats so that they were undernourished increased the histone acetylation and HIF-1α binding levels in the proximal promoter region of ET-1, up-regulating the expression of ET-1, and this continued for 6 weeks after birth of the offspring. The authors speculate that that this intrauterine growth retardation could cause varying degrees of PAH later in life.

Generally, increased levels of histone acetylation are associated with increased transcriptional activity, whereas decreased levels of acetylation are correlated with suppressed gene expression. These data show that the open chromatin domains marked by histone H3 and H3K9/18 acetylation at the proximal promoter of ET-1 in IUGR rats are essential for transcription. Up-regulated ET-1 protein expression in PVEC from IUGR hypoxia rats is closely associated with the presence of increased acetylated H3 histones.

### Biased signalling in the ET pathway

Pharmacology is undergoing a revolution in understanding the mechanism of ‘biased signalling’ via GPCRs. It was originally thought that ligands binding to a receptor would equally activate the G-protein pathway to produce a physiological response such as vasoconstriction (such as ET-1 acting on an ET_A_ receptor) as well as activating the β-arrestin pathway, which eventually leads to desensitization, receptor internalization and ‘silencing’ of the pathway. It is now clear that some ligands are biased to one pathway over the other and secondly, rather than silencing, β-arrestin can activate alternative signalling pathways, some of which may be pathophysiological leading to longer-term signalling responses such as migration and proliferation.

Both ET receptor subtypes follow an β-arrestin and dynamin/clathrin-dependent mechanism of internalization, but it has been established that ET_A_ receptors are recycled to the plasma membrane for further signalling while ET_B_ receptors are targeted to lysosomes and degraded (Bremnes *et al*., 2000). In epithelial ovarian cancer, activation of ET-1/ET_A_ receptor signalling is linked to many tumour-promoting processes including proliferation, angiogenesis, invasion and metastasis. NF-κB is an important signalling molecule in immunity, inflammation and cancer and β-arrestin is required for ET–1-induced NF-κB activation (Cianfrocca *et al*., 2014). ET-1 promoted podocyte migration via ET_A_ receptors and increased β-arrestin-1, sustaining renal injury, a pathogenetic pathway that can affect podocyte phenotype in proliferative glomerular disorders (Buelli *et al*., 2014). β-Arrestin-1 has also been found to be a nuclear transcriptional regulator of ET–1-induced β-catenin signalling, an important mechanism for controlling cell division and progression of epithelial ovarian cancer and necessary for epigenetic modification, such as histone acetylation, and gene expression (Rosanò *et al*., 2009; 2013a[Bibr b126],[Bibr b127]). In addition, these effects are blocked by ET receptor antagonists and support a role for ET_A_-mediated/β-arrestin-1 facilitating inter-protein interaction in invasive and metastatic behaviour of ovarian cancer.

### Biased ET ligands?

Agonists that are biased towards β-arrestin signalling for parathyroid hormone and angiotensin AT_1_ receptors have been identified. G-protein pathway-selective agonists have been identified for nicotinic acid (nomenclature revised by NC-IUPHAR to hydroxycarboxylic acid) and μ-opioid receptors (Luttrell, 2014). The race is now to determine whether such strategies can be exploited therapeutically.

Do biased ligands (ligands that binding to the same receptor but activate different signalling pathways) exist for ET receptors? The study by Compeer *et al*. (2013) already mentioned suggested ET-1 and ET-2 initiate and maintain vasoconstriction by different downstream mechanisms. Biased signalling can be identified by comparing the affinities of ligands in β-arrestin recruitment assays with a G–protein-mediated response such as vasoconstriction (Maguire *et al*., 2012). In this study the rank order of potency for β-arrestin recruitment at the ET_A_ (ET-1 ≥ ET-2 > > ET-3) and ET_B_ (ET-1 = ET-2 = ET-3) receptors was as expected and there was no obvious major differences in potency of ETs when comparing with G–protein-mediated constrictor assays in human vessels. However, at the ET_A_ receptor sarafotoxin S6b was a partial agonist in β-arrestin recruitment, but a full agonist in causing constriction, suggesting the possibility of biased ligands. Such a bias could have been selected for during evolution by prolonging the effects of envenomation of the mammalian prey. While bosentan displays no selectivity for ET_A_ over ET_B_ receptors in radioligand binding and G-protein functional assays, unexpectedly, it was significantly more effective an inhibitor of β-arrestin recruitment mediated by ET_A_, compared with the ET_B_ receptors (Maguire *et al*., 2012). The result for bosentan is intriguing as many of the detrimental actions of ET-1, particularly in cancer, may use the β-arrestin pathway, and this suggests the potential to block a deleterious pathway while preserving activation of a beneficial pathway.

## The next 25 years – quo vadis?

Since the discovery of ET, intense medicinal chemistry programmes have identified receptor antagonists, ET_B_ receptor agonists and inhibitors of the key synthetic enzyme, ECE-1. Both mixed ET_A_/ET_B_ and ET_A_ selective receptor antagonists have become established in the treatment of PAH, while NEP/ECE inhibitors such as SLV306 have promise as an alternative to receptor blockade and IRL1620 and other ET_B_ receptor ligands have potential in improving cancer therapy.

All of these are approaches that have exploited low MW compounds. Over 50 therapeutic monoclonal antibodies have been approved for clinical use, but none yet against a GPCR target, emphasizing the technical challenge. Endomab-B1, a monoclonal antibody has recently been reported to bind with subnanomolar affinity for the ET_B_ receptor, competed with ET-1 binding with greater efficacy than BQ788, and functions as an antagonist to block the ET–1-induced IP_3_-calcium signalling pathway (Allard *et al*., 2013). Whether this antibody has clinical applications remains to be discovered.

## Abbreviations

ECE-1: endothelin-converting enzyme-1
ECE-2: endothelin-converting enzyme-2
ELDP: endothelin-like domain peptide
ET: endothelin
HIF-1α: hypoxia inducible factor-1α
NEP: neutral endopeptidase
PAH: pulmonary arterial hypertension
